# Feasibility of school-based health education intervention to improve the compliance to mass drug administration for lymphatic Filariasis in Lalitpur district, Nepal: A mixed methods among students, teachers and health program manager

**DOI:** 10.1371/journal.pone.0203547

**Published:** 2018-09-14

**Authors:** Prativa Karki, Yayi Suryo Prabandari, Ari Probandari, Megha Raj Banjara

**Affiliations:** 1 Department of Public Health, Universitas Gadjah Mada, Yogyakarta, Indonesia; 2 Department of Public Health, Faculty of Medicine, Universitas Sebelas Maret, Indonesia; 3 Center for Tropical Medicine, Universitas Gadjah Mada, Yogyakarta, Indonesia; 4 Central Department of Microbiology, Tribhuvan University, Kathmandu, Nepal; B P Koirala Institute of Health Sciences, NEPAL

## Abstract

**Background:**

Ensuring reduction in transmission of lymphatic Filariasis (LF) and addressing the compliance of people to mass drug administration (MDA) has led to renewed efforts in the field. School-based health education (SBHE) intervention, considered a cost-effective strategy with potential to reach the wider public through young people, was adopted as a strategy for social mobilization. This study assessed SBHE perceptions, implementation barriers, and factors in the supporting environment as well as its efficiency to successfully change LF MDA-related knowledge and practice.

**Methods:**

This mixed methods study was conducted in four sites of Lalitpur district, Nepal. Classroom-based interactive health education sessions were used as the main intervention strategy in the study. In total, 572 students were assigned to intervention and control groups. Questionnaires were distributed before and after the intervention. Mann-Whitney and McNemar tests were used for analysis. Focus-group discussions with teachers and students and in-depth interviews with the district LF program manager as well as Education Office and school management authorities were conducted. Qualitative thematic analysis approach was adopted.

**Results:**

Intervention curriculum played a significant role in increasing children’s knowledge and practice (p<0.001). Barriers for school-based interventions were budget constraints, human resource deficiencies, lack of opportunities to conduct practical classes under the curriculum, and lack of collaboration with parents. Supportive factors were training provision, monitoring and evaluation practice, adequate facilities and equipment, positive parental attitudes, presence of interested teachers and students, and prioritization by program implementers.

**Conclusion:**

Effective program planning practices such as proper fiscal management, human resource management, training mechanisms, and efforts to promote practical classes and coordination with parents are required to develop and institutionalize the intervention. Effective learning and a supportive school environment appear to be important components to support implementation. The SBHE intervention is a feasible and promising intervention for accelerating compliance towards MDA to eliminate LF.

## Introduction

In Nepal, Lymphatic Filariasis (LF) is endemic in two-thirds of its districts accounting risk for 60% of the total population [1,2]. National LF prevalence shows 13% prevalence ranging from <1% to 39% [1–4]. Mass drug administration (MDA) campaigns are a primary preventive strategy which involves combined dose of two medicines Diethylcarbamazine (DEC) and albendazole (ABZ) given annually continued for 4 to 6 years to an entire risk population that will help for the interruption of LF transmission and compliance to it is must for the prevention and elimination of LF [5–8]. The evidence indicates that compliance to MDA drugs can reduce the risk of developing the disease [9,10]. Children are particularly vulnerable group among the at-risk population, as LF is acquired at a young age. Facilitating compliance to MDA drugs in children at an early age can help them to prevent LF in future [11–15]. Therefore, awareness and compliance among the children too is necessary along with the adult population.

Studies show that transmission of the disease can be disrupted through annual treatment with anti-filarial drugs for an estimate of at least > 90% of the population [16]. However, awareness activities appear ineffective at achieving and sustaining consistent levels of compliance to MDA programs especially in the highly populous areas [1,2]. There have been numerous awareness campaigns to promote compliance such as several advocacy campaigns, community and social mobilization activities are carried out every year during MDA at various level (national, regional, implementation unit, and community) [17]. But the persistent suspicion of drugs and fears concerning their potential side effects represent a huge barrier to the uptake of MDA drugs, which remains one of the biggest challenges for elimination of LF in Nepal [4]. Intensive health education is of utmost importance to raise awareness in people to facilitate an increase towards maximum compliance levels [2]. The research suggests if appropriate awareness strategies are targeted at a specific population with an appropriate health education message then it can help to facilitate a change [10,18]. If health education is given to children at a young age, it will have an influence on their attitude and enhance their knowledge and skills [19].

Since MDA drugs are distributed to an entire group of people regardless of their age except for <2 years children and pregnant women, targeting school children who occupy one-third of population could provide a sustainable information flow to larger population as they can act as “messengers” to their family and community [20–23]. Moreover, with a huge enrollment of students at school and for a longer period of their lives, school can act as the important institution to provide the children with reliable information and help for the information flow to a wider community [24–26]. These findings suggest that educating and creating awareness among school children is endorsed as a robust strategy for many diseases and their prevention in a broader context. School-based health education (SBHE) interventions are considered as a cost-effective strategy to improve and sustain compliance among students and community members [25].

The motivation of this study is based on the PRISM (Practical, Robust Implementation Sustainability Model) domains to examine how an evidence-based program like SBHE interacts with recipients in order to influence program implementation and continuation [27]. The questions addressed in this study examines the feasibility of SBHE intervention to assist an LF MDA intervention and increase its coverage and facilitate an impact on the knowledge and behavioral change for LF MDA among school children. We measured the perception of the stakeholders, identified barriers, and investigated the supporting environment for the interventions implementation.

## Materials and methods

### Research type and design

This mixed method study utilized the convergent design approach to provide both a qualitative and quantitative picture. This study will involve the separate collection and analysis of quantitative and qualitative data. The results thus will be merged to produce two interpretations for both statistical and thematic qualitative results to key assessment of feasibility of intervention. In this study, a quasi-experimental design was utilized, which included an intervention and a control group and a pre-post assessment to observe the effect of the intervention. The selected sites in study area were assigned to intervention (two sites) and control group (two sites) by lottery method. We simultaneously illustrated SBHE perceptions, implementation barriers, and the supporting operational environment based on five key stakeholder groups: students, teachers, the LF focal person, the school principal, and an officer from education office.

### Study settings and time

The study was conducted in four selected sites of the highly populous Lalitpur District in Kathmandu Valley, given that it has a greater prevalence of antigenemia (antigens detection in peripheral blood for mapping and monitoring of LF elimination programs) and did not qualify for the elimination of LF in pre-TAS (Transmission Assessment Survey) in 2014 and 2015 [17] [28]. Intervention and field data collection was carried out from February 2017 to April 2017. The district was one of the three districts in the capital city covering an area of 385 km^2^ with a dense population of 468,132 [28]. There were 19 village development committees (VDCs) and 4 VDCs were selected randomly for this study. There were no private schools in these VDCs so we included all the schools with altogether 7 schools as study sites. The primary school level does not have a health education curriculum, so we excluded those schools, and selected three lower secondary and four secondary government schools as our study area.

### Intervention

The SBHE intervention consists of classroom-based interactive health education sessions targeted at school children with the intention to enhance their knowledge and practices regarding LF MDA. An intervention manual was developed to do so, based on information from existing LF IEC materials that were reviewed and approved by a panel of experts (Table 1).

**Table 1:** Description of components of intervention manual and its implementation strategies.

Description	Intervention components		
	Education components		Interactive components
*Delivery session*	**Session 1** Lymphatic Filariasis (LF) Causes Sign/symptoms Transmission Identifying at-risk population LF prevention	**Session 2** MDA program Benefits of MDA drugs and its compliance Non-compliance factors Rumours about LF and MDA	Group work Games Quiz Drama
	**Implementation strategies**		
*Frequency*	Two times in a school		
*Duration*	40–60 minutes per session		
*Intensity*	Basic information to increase knowledge on LF MDA, address benefit of MDA & rumours		
*How is it implemented*	Classroom sessions by researcher with the help of assistant		
*When is it implemented*	Before MDA program in March in intervention school		
*By whom is it implemented*	Research team		

The sessions covered by the manual consisted of both educational and interactive components (i.e., group work, drama, quizzes, and games). The educational component consisted of sessions on LF (i.e., causes, signs/symptoms, risk factors, and prevention) and MDA (benefits of MDA, and non-compliance factors to MDA) whereas the interactive component consisted of group work, role play, quizzes, and games. At the end of each session, students were asked to develop short key messages and were encouraged to share these messages with their family members and friends.

The sessions were delivered by the principal investigator with the assistance from other team members. Each session was approximately 40–60 minutes (as per the time allocated to one lecture in school) in duration. The intervention was delivered two times before the MDA program in each school with a total of two visits in each school. The primary outcome of the intervention was the assessment of the level of knowledge and practice.

### Sample size

The total population from seven selected schools was the study population. The list of the number of students from each of the selected seven schools was obtained from the school registry of each respective school, which resulted in a total number of 611 students from grade 6 to 9. Students from grade 10 were excluded due to their unavailability for post-assessment as they would not be available in the schools after the national level centralized examination. All the students who were interested and present on the day of data collection as well as had both parental consent and student assent, were included as participants in the study. However, considering the possibility of absentee students and refusal or drop out in the middle of the study, a non-response rate of 5–10% was acknowledged. Accounting for missing data, we obtained a total sample size of 572. However, due to the loss of 34 students (6.3%) on the follow up, the final sample size was 538 participants in the end line survey.

For the qualitative study, we conducted six focus-group discussion (FGD) with 8 to 10 participants among teachers and students independently in each intervention school, along with one-on-one in-depth interviews (IDI) with school management in each of the intervention schools, the LF focal person from the District Public Health Office, and an officer from the District Education Office. The discussion and interview were conducted with the related stakeholders depending on their availability.

### Data collection and research instrument

The data were collected using qualitative FGD guidelines, interviews, and assessment questionnaires. The questionnaires and guidelines were pilot-tested and then revised before use. Those pilot-tested questionnaires and guidelines were not used for the study. For both the quantitative and qualitative method, a Nepali (local language) version of the structured questionnaire was used to collect data by the research team.

The assessment questionnaire consisted of nine questions on LF MDA knowledge and one question assessing the person’s participation in previous MDA. Self-administered questionnaires were distributed to each participant before and after the intervention to collect the demographics of each participant and their information concerning their knowledge and practice towards mass drug administration of LF. FGDs and IDIs were conducted by the research team to obtain insight into the perceptions of relevant parties, identify barriers to implementation, and understand the influence of the supportive environment on SBHE implementation.

The research assistants were trained before the survey to orient them to the study instruments in order to ensure completeness during the field survey. The questionnaire used at baseline was used again at the end-line with necessary adjustments. The research team was recruited based on the criteria that the researchers had been from a public health field. The questionnaire was developed based on a previous study [2,25,29].

### Data analysis

Quantitative data were analyzed using Stata software version 13 for Windows. Each filled questionnaire was checked for completeness every time after data collection. For any missed variables and errors, computer frequencies were used and the identified errors were corrected by revising the original questionnaire. LF MDA-related knowledge was assessed using nine items. The responses to the information were either stated as ‘1 = yes’, ‘2 = no’, and ‘3 = don’t know’ and were re-categorized into two groupings of ‘no’ and ‘don’t know’ as 0 and ‘yes’ as 1 and for those participants with multiple responses, that is participants who mentioned two or more answers, they were labeled as knowledgeable. Counting every correct answer score of 1 resulted in producing a total score of 9 points. This scoring system applied was based on previous studies [30–32]. However, to measure compliance, we used one item for which every response to question “Did you take the last dose of MDA drugs?” was recorded as either 1 = yes and 2 = no, and given a score of 1 indicating MDA compliance. A Mann-Whitney test and a McNemar test was used to examine the level of knowledge scores and practice of drug uptake before and after the intervention [19].

Qualitative data were analyzed using the thematic analysis approach where coding was performed first, in order to develop the themes from raw data. To assess consistency, the author and research assistant independently coded each passage, and this was developed in a matrix form for the analysis. During coding, relevant segments of text were labeled as either pertaining to perceptions, barriers, or a supportive operational environment as themes. Through the identification of the important themes within the understanding of participants, coding was done before the interpretation.

### Research ethics

The ethical application and consent procedure of this study was reviewed and the grant of approval for the study was obtained from the Ethical Review Board of Nepal Health Research Council (NHRC). A formal approval letter from the Epidemiology and Disease Control Division (EDCD), the District Education Office (DEO), and respective schools under study were obtained for data collection. Letters were distributed to all the schools in the study requesting for their cooperation and participation. Consent forms were given to participants and were obtained prior to the study. Students’ assent forms and informed parental consent forms were given to participants to explain the study in advance and request them to obtain written consent. Students who received the consent from their parents/ guardians, were provided with detailed explanations of this study, and they were requested to sign the informed consent forms. Participants were informed participation was voluntary, that their information would be confidential, and their rights to refuse participation at any time during the study.

## Results

There were 572 participants at baseline (intervention = 289 and control = 283) and 538 (intervention = 276 and control = 262) at end-line. The mean age of participants in the study population was 13.7 years (± SD 1.5 years) with the majority being from the Tamang ethnicity and followers of Hinduism. When assessing the knowledge score, we found significant improvements in the mean score of the participants in the intervention group whereas in the control group, the mean scores were not statistically different at any level smaller than 2.03% (Table 2). There was a change in the mean of the knowledge score of the participants with a 2.24 mean change that was attributed to the effect of the intervention.

**Table 2:** Effect of intervention on mean knowledge score.

Group	Baseline		End line		Change	p-value*
	Mean	SD	Mean	SD		
**Intervention**	3.03	1.50	6.15	1.42	3.12	0.000
**Control**	3.52	1.51	4.40	1.54	0.98	0.020
**Difference**			1.85		2.24	

*Mann-Whitney test to compare between intervention and control: Significance at 0.05

Of the students targeted by the SBHE intervention with the aim of effecting a change in their practice, primarily, in their intake of drugs as the major change, there was a significant change (p < 0.001) as 26 percent of the participants reported a change in their practice of drug uptake (Table 3). Whereas in the control group there was no significant changes (p = 0.748) on the baseline and end line MDA drugs uptake practice.

**Table 3:** Effect of intervention on practice of drug uptake.

Practice of drug uptake	Baseline (%)	End line (%)	Change	P-value
**Intervention**	69.20	89.49	20.29	<0.0001
**Control**	57.59	51.90	-5.69	0.748
**Difference**		37.59	25.98	

*Significance at 0.05 (McNemar test)

The results from the study identified the primary reason for abstaining from taking the drugs by the participants and fear of side effects and parents forbidding the children to take the drugs were reported to be the most important factors for not taking drugs (Table 4). Results also indicated that the implementation of the intervention reduced fear of taking drugs and parents’ disapproval to take drugs (p<0.001).

**Table 4:** Reasons for not taking MDA drugs.

Reason for not taking MDA drugs	Baseline (n = 211)				End line (n = 201)				P-value*
	C_B_		I_B_		C_E_		I_E_		
	n	%	n	%	n	%	n	%	
**Not at home**	20	16.52	19	21.11	37	29.36	10	34.48	0.007
**Fear of adverse events**	70	57.85	43	47.77	79	62.69	9	31.03	0.000
**Health worker did not deliver drugs**	13	10.74	14	15.55	19	15.07	2	6.89	0.002
**Parents didn’t allow**	55	45.45	64	71.11	31	24.60	3	10.34	0.000
**Dislike medicine**	13	10.74	15	16.66	10	7.93	6	20.68	0.305
**Don’t have any reason**	24	19.83	13	14.44	23	18.25	3	10.34	0.034

C_B_ = Control group at baseline, I_B_ = Intervention group at baseline, C_E_ = Control group at end line, I_E_ = Intervention group at end line

The findings from qualitative study are summarized in Table 5, which shows the minor themes for each of the major themes generated as well as illustrative quotes from participants that correspond to the minor themes. Certain barriers and factors in the supportive operational environment represented different sides of a common factor (e.g., insufficient training for the teachers versus provision of training to teachers). Other barriers and factors in the supportive operational environment were independent and unique (e.g., lacking opportunity to conduct practical classes under the curriculum, priority to health education related program over other programs by stakeholders, etc.).

**Table 5:** Perception, barriers and supportive operational environment of SBHE implementation.

Themes	Illustrative quotes
Perception	
School management perceive the intervention to be positive	*SM*:*”If such SBHE is organized from time to time positive messages about health would spread and overall the community would be healthy”*
Belief of students to have knowledge and in self-decision making	*Student*:*”can decide to take or not to take drugs and also convince other family members to do so*.*”*
Relevance of the intervention	*Officer from Education Office*:*”Making students aware and through them to their parents even teachers will be aware*, *will help to make people aware and take positive action to it*.*”*
Consideration for the teacher’s skill while implementing intervention	*Teachers*:*”Yes*, *the ability of teachers to comprehend the knowledge should be considered*. *If they can comprehend nicely then they can deliver the session to students nicely in a way they can understand*.*”*
Implementation barrier	
Parents frustration over intervention due to lack of collaboration	*Teachers*:*”parents complaining about lack of curriculum studies and more of extra studies”*
Lack of money for program conduction	*SM*:*”First thing is financial barrier as for any programme to be conducted first thing is we need money and we don’t have enough money if we have to conduct such programme*.*”*
Time constraints	*LF focal person*:*”I think the time allocation for school-based programme is very less to bring change in knowledge and practice…*..*”*
Supportive operational environment	
Priority of the stakeholders	*SM*:*”If a program has to be prioritized between sports and other health related program*, *I would give priority to health related practical or theoretical programs because it will help for the mental growth of children*.*”*
Mechanism for M& E practice	*LF focal person”we have monitoring and evaluation mechanism*. *We do it through questionnaire*, *ask students and have interaction with teachers”*
Adequate facilities and equipment	*Officer from Education office*:*”Yes*, *we have enough expertise and information available and if we lack also we upgrade it”*

## Discussion

The results of the study were able to identify that effective health education has the capability to not only result in the acquisition of knowledge but also bring about desired changes in practice [19]. Documenting a change in knowledge and practice is important to prove that there was some gain in the children’s knowledge and that their actions resulted in positive change, which is illustrated by the positive impact of the SBHE intervention [24,33]. The present study also demonstrates that knowledge and practice regarding LF MDA among the school children were low before the implementation of the intervention, but after its implementation, results showed significant increases in mean LF MDA knowledge (3.03 to 6.15) and practice (69.78% to 89.6%) for children in the intervention group. The results of other studies also showed the similar impact to the observed increase in the post-test score of the participants as a result of the intervention [30,32–35].

Our findings also showed school-based intervention and health curriculum have increased the level of children’s knowledge scores, which had its impact on the practice of drug compliance compared to the control curriculum (p<0.001) [24,25,33]. These findings from the study were also supported from the FGDs with students which corroborates our reported data.

This study also revealed some of the barriers for MDA drug uptake (Table 5). During the baseline survey, 71.11% of the participants reported that they did not take the drugs due to restrictions from their parents and 47.77% reported that they had fears concerning the side effects of these drugs. However, significant improvements concerning barriers for drug uptake were observed at follow up. Yet, no significant differences were observed regarding barriers in the control group. These findings demonstrate that the intervention was also able to help remove these barriers towards drug uptake.

We also found that change in practice after the educational intervention can be sustained in the long term, which was also supported by the qualitative findings from the study as the stakeholders perceive the program to be sustainable [24]. We found that when the perception and understanding of the target population are incorporated in an intervention, it leads the intervention towards success [36,37].

We also identified issues in program planning as the most important implementation barrier as there was no proper budget allocation causing budget deficits, improper training methods, and lack of collaboration, which were also identified in other studies [33,38]. The study also reported that having a positive supportive factors was found to be significant as their presence would have influence over the implementation of the intervention [38,39]. The findings from this study will serve as a resource for the implementation and conducting of successive interventions and also support the fact that trying to convey a message through students will have an impact on their knowledge and practice.

Though there have been few studies to show the impact of school health [40,41]. There is very little research concerned with the perception of stakeholders concerning interventions, barriers toward the implementation of interventions, and the existence of supporting operational factors in interventions. Despite the fact that there are some limitations regarding recall bias, desirability bias, validity, generalizability and the influence of extraneous factors, the current study provides new and important findings concerning the feasibility of school-based health educational interventions to foster compliance towards MDA of LF among school children.

## Conclusion

Our study concluded that the SBHE intervention could improve the compliance to mass drugs administration for lymphatic Filariasis. The intervention is feasible, with some consideration of facilitating factors particularly in planning and executing the program. Effective program planning practices such as proper fiscal management, human resource management, training mechanisms, and efforts to promote practical classes and coordination with parents are required to develop and institutionalize the intervention. Effective learning and a supportive school environment appear to be important components to support implementation.

## Supporting information

S1 Table Description of components of intervention manual and its implementation strategies. (DOCX)

S2 Table Effect of intervention on mean knowledge score. (DOCX)

S3 Table Effect of intervention on practice of drug uptake. (DOCX)

S4 Table Reasons for not taking MDA drugs. (DOCX)

S5 Table Perception, barriers and supportive operational environment of SBHE implementation. (DOCX)

S1 File Supplementary file. (DOCX)
