# Supplementary material for: Feasibility of school-based health education intervention to improve the compliance to mass drug administration for lymphatic Filariasis in Lalitpur district, Nepal: A mixed methods among students, teachers and health program manager
Source: PLoS One. 2018 Sep 14;13(9):e0203547. doi: 10.1371/journal.pone.0203547 (PMC6138383; doi:10.1371/journal.pone.0203547)
Supplement: S1 Table — (DOCX) [file pone.0203547.s001.docx]

**Table 1. Description of components of intervention manual and its implementation strategies**

| **Description** | **Intervention components** | | |
| --- | --- | --- | --- |
|  | **Education components** | | **Interactive components** |
| *Delivery session* | **Session 1**  Lymphatic Filariasis (LF)   - Causes - Sign/symptoms - Transmission - Identifying at-risk population   LF prevention | **Session 2**  MDA program   - Benefits of MDA drugs and its compliance   Non-compliance factors   - Rumours about LF and MDA | Group work  Games  Quiz  Drama |
|  | **Implementation strategies** | | |
| *Frequency* | Two times in a school | | |
| *Duration* | 40-60 minutes per session | | |
| *Intensity* | Basic information to increase knowledge on LF MDA, address benefit of MDA & rumours | | |
| *How is it implemented* | Classroom sessions by researcher with the help of assistant | | |
| *When is it implemented* | Before MDA program in March in intervention school | | |
| *By whom is it implemented* | Research team | | |
