# Supplementary material for: Feasibility of school-based health education intervention to improve the compliance to mass drug administration for lymphatic Filariasis in Lalitpur district, Nepal: A mixed methods among students, teachers and health program manager
Source: PLoS One. 2018 Sep 14;13(9):e0203547. doi: 10.1371/journal.pone.0203547 (PMC6138383; doi:10.1371/journal.pone.0203547)
Supplement: S2 Table — (DOCX) [file pone.0203547.s002.docx]

| Group | Baseline |  | End line |  | Change | p-value* |
| --- | --- | --- | --- | --- | --- | --- |
|  | **Mean** | **SD** | **Mean** | **SD** |  |  |
| Intervention | 3.03 | 1.50 | 6.15 | 1.42 | 3.12 | 0.000 |
| Control | 3.52 | 1.51 | 4.40 | 1.54 | 0.98 | 0.020 |
| Difference |  |  | 1.85 |  | 2.24 |  |

**Table 2. Effect of intervention on mean knowledge score**

*Mann-Whitney test to compare between intervention and control: Significance at 0.05
