# Supplementary material for: Feasibility of school-based health education intervention to improve the compliance to mass drug administration for lymphatic Filariasis in Lalitpur district, Nepal: A mixed methods among students, teachers and health program manager
Source: PLoS One. 2018 Sep 14;13(9):e0203547. doi: 10.1371/journal.pone.0203547 (PMC6138383; doi:10.1371/journal.pone.0203547)
Supplement: S3 Table — (DOCX) [file pone.0203547.s003.docx]

**Table 3. Effect of intervention on practice of drug uptake**

| Practice of drug uptake | Baseline (%) | End line (%) | Change | P-value |
| --- | --- | --- | --- | --- |
| Intervention | 69.20 | 89.49 | 20.29 | <0.0001 |
| Control | 57.59 | 51.90 | -5.69 | 0.748 |
| Difference |  | 37.59 | 25.98 |  |

*Significance at 0.05 (McNemar test)
