# Supplementary material for: Feasibility of school-based health education intervention to improve the compliance to mass drug administration for lymphatic Filariasis in Lalitpur district, Nepal: A mixed methods among students, teachers and health program manager
Source: PLoS One. 2018 Sep 14;13(9):e0203547. doi: 10.1371/journal.pone.0203547 (PMC6138383; doi:10.1371/journal.pone.0203547)
Supplement: S4 Table — (DOCX) [file pone.0203547.s004.docx]

**Table 4. Reasons for not taking MDA drugs**

| Reason for not taking MDA drugs | Baseline (n=211) | | | | End line (n=201) | | | | P-value* |
| --- | --- | --- | --- | --- | --- | --- | --- | --- | --- |
|  | **C_B_** | | **I_B_** | | **C_E_** | | **I_E_** | |  |
|  | **n** | **%** | **n** | **%** | **n** | **%** | **n** | **%** |  |
| Not at home | 20 | 16.52 | 19 | 21.11 | 37 | 29.36 | 10 | 34.48 | 0.007 |
| Fear of adverse events | 70 | 57.85 | 43 | 47.77 | 79 | 62.69 | 9 | 31.03 | 0.000 |
| Health worker did not deliver drugs | 13 | 10.74 | 14 | 15.55 | 19 | 15.07 | 2 | 6.89 | 0.002 |
| Parents didn’t allow | 55 | 45.45 | 64 | 71.11 | 31 | 24.60 | 3 | 10.34 | 0.000 |
| Dislike medicine | 13 | 10.74 | 15 | 16.66 | 10 | 7.93 | 6 | 20.68 | 0.305 |
| Don’t have any reason | 24 | 19.83 | 13 | 14.44 | 23 | 18.25 | 3 | 10.34 | 0.034 |

C_B_=Control group at baseline, I_B_=Intervention group at baseline, C_E_= Control group at end line, I_E_= Intervention group at end line
