# Supplementary material for: Feasibility of school-based health education intervention to improve the compliance to mass drug administration for lymphatic Filariasis in Lalitpur district, Nepal: A mixed methods among students, teachers and health program manager
Source: PLoS One. 2018 Sep 14;13(9):e0203547. doi: 10.1371/journal.pone.0203547 (PMC6138383; doi:10.1371/journal.pone.0203547)
Supplement: S5 Table — (DOCX) [file pone.0203547.s005.docx]

**Table 5. Perception, barriers and supportive operational environment of SBHE implementation**

| **Themes** | **Illustrative quotes** |
| --- | --- |
| Perception | |
| School management perceive the intervention to be positive | *SM:”If such SBHE is organized from time to time positive messages about health would spread and overall the community would be healthy”* |
| Belief of students to have knowledge and in self-decision making | *Student:”can decide to take or not to take drugs and also convince other family members to do so.”* |
| Relevance of the intervention | *Officer from Education Office:”Making students aware and through them to their parents even teachers will be aware, will help to make people aware and take positive action to it.”* |
| Consideration for the teacher’s skill while implementing intervention | *Teachers:”Yes, the ability of teachers to comprehend the knowledge should be considered. If they can comprehend nicely then they can deliver the session to students nicely in a way they can understand.”* |
| Implementation barrier | |
| Parents frustration over intervention due to lack of collaboration | *Teachers :”parents complaining about lack of curriculum studies and more of extra studies”* |
| Lack of money for program conduction | *SM:”First thing is financial barrier as for any programme to be conducted first thing is we need money and we don’t have enough money if we have to conduct such programme.”* |
| Time constraints | *LF focal person:”I think the time allocation for school-based programme is very less to bring change in knowledge and practice…..”* |
| Supportive operational environment | |
| Priority of the stakeholders | *SM:”If a program has to be prioritized between sports and other health related program, I would give priority to health related practical or theoretical programs because it will help for the mental growth of children.”* |
| Mechanism for M& E practice | *LF focal person”we have monitoring and evaluation mechanism. We do it through questionnaire, ask students and have interaction with teachers”* |
| Adequate facilities and equipment | *Officer from Education office:”Yes, we have enough expertise and information available and if we lack also we upgrade it”* |
