# Supplementary material for: Feasibility of school-based health education intervention to improve the compliance to mass drug administration for lymphatic Filariasis in Lalitpur district, Nepal: A mixed methods among students, teachers and health program manager
Source: PLoS One. 2018 Sep 14;13(9):e0203547. doi: 10.1371/journal.pone.0203547 (PMC6138383; doi:10.1371/journal.pone.0203547)
Supplement: S1 File — (DOCX) [file pone.0203547.s006.docx]

**Pre-Intervention Questionnaire**

**Feasibility of School-based health education intervention for compliance of mass drug administration of Lymphatic Filariasis.**

**Instruction:** You are requested to answer the following questions. Encircle the appropriate answer. You can encircle more than one answer. The answers obtained will be used only for the study purpose and will be kept confidential.

**Objectives**: The objective of this assessment is to assess the knowledge and practice of Lymphatic Filariasis (LF) and Mass drug administration (MDA) among lower secondary and secondary level students from government schools in selected sites of Lalitpur district, Nepal.

**GROUP A: Socio-demographic characteristics**

1. Code no……………………………………….…
2. Age: (completed years)………………………….
3. Gender: ……………………………………….....
4. Class: ……………………………………………
5. Ethnic group:
6. Brahmin 1
7. Chhetri 2
8. Newar 3
9. Tamang 4
10. Gurung 5
11. Others (specify)……….... 6
12. Religion:
13. Hindu 1
14. Buddhist 2
15. Christian 3
16. Muslim 4
17. Others (specify)…………… 5

**GROUP B: Household characteristics**

1. How many members do you have in your family? ………………………………...
2. What is the education level of your father?

a) Literate 1

b) Illiterate 2

c) Primary level 3

d) Secondary level 4

e) College/ University 5

1. What is the education level of your mother?

a) Literate 1

b) Illiterate 2

c) Primary level 3

d) Secondary level 4

e) College/ University 5

1. What is your father’s occupation?

a) Agriculture 1

b) Service 2

c) Business 3

d) Wage/labour 4

e) Housework 5

f) Others (specify)…………………………. 6

1. What is your mother’s occupation?

a) Agriculture 1

b) Service 2

c) Business 3

d) Wage/labour 4

e) Housework 5

f) Others (specify)…………………………. 6

**GROUP C: Knowledge and practice on LF and MDA**

1. Have you heard about Lymphatic Filariasis or elephantiasis?
2. Yes 1
3. No 2
4. From where did you heard/learn about the disease?
5. People distributing MDA 1
6. Relatives 2
7. Friends 3
8. Written materials (newspaper, posters) 4
9. Television Advertisements 5
10. Schools/Teachers 6
11. Others (specify)……………………… 7
12. What kind of microorganism causes Lymphatic Filariasis?
13. Parasite 1
14. Viruses 2
15. Bacteria 3
16. Fungi 4
17. Don’t Know 5
18. How is Lymphatic Filariasis spread? (Multiple choice)
19. Airborne (sneezing, coughing, etc.) 1
20. Food 2
21. Genetically inherited 3
22. Mosquitoes 4
23. Witch 5
24. Don’t know 6
25. Others (specify)…………………. 7
26. Lymphatic Filariasis is mostly seen in countries that are in the tropical or sub-tropical locations.
27. Yes 1
28. No 2
29. Don’t know 3
30. An individual can contract Lymphatic Filariasis after just one bite
31. Yes 1
32. No 2
33. What are the preventive measures of Lymphatic Filariasis? (Multiple choice)
34. Mosquito net 1
35. Fumigation 2
36. Isolation from patient of LF 3
37. Sanitation 4
38. Don’t know 5
39. Others(specify)……………………………………………………………….....6
40. There is no treatment for Lymphatic Filariasis
41. Yes 1
42. No 2
43. Don’t know 3
44. The signs and symptoms of Lymphatic Filariasis include: (Multiple Choice)
45. Body aches and pain 1
46. Fever 2
47. Lymphatic edema 3
48. Hydrocele 4
49. All of the above 5
50. Don’t Know 6
51. Once a person has the swelling of limbs, he/she cannot control the progression of the condition
52. Yes 1
53. No 2
54. The medicines used in MDA prevent LF
55. Yes 1
56. No 2
57. Did you take MDA drugs in last round of MDA campaign in the month of Feb-March? **(If yes, skip to Ques. 14)**
58. Yes 1
59. No 2
60. Don’t know 3
61. If no, why didn’t you take MDA drugs? (Multiple Choice)
62. Not at home 1
63. Fear of adverse events 2
64. Health worker did not deliver drugs 3
65. Parent’s didn’t allow 4
66. Dislike medicine 5
67. Don’t have any reason 6
68. Have you had any side effects after you took the drugs?
69. Yes 1
70. No 2
71. If yes, what were the side effects? ( Multiple choice)
72. Dizziness 1
73. Headache 2
74. Stomach ache
75. Nausea and vomiting 3
76. Fever 4
77. Others (specify)……………………………. 5
78. Did you have any treatment of side effect after?
79. Yes 1
80. No 2
81. No response 3

**Thank You!**

**Post-intervention Questionnaire**

**Feasibility of School-based health education intervention for compliance of mass drug administration of Lymphatic Filariasis.**

**Instruction:** You are requested to answer the following questions. Encircle the appropriate answer. You can encircle more than one answer. The answers obtained will be used only for the study purpose and will be kept confidential.

**Objectives**: The objective of this assessment is to assess the knowledge and practice of Lymphatic Filariasis (LF) and Mass drug administration (MDA) among lower secondary and secondary level students from government schools in selected sites of Lalitpur district, Nepal.

**GROUP A: Socio-demographic characteristics**

1. Code no……………………………………….…
2. Age: (completed years)………………………….
3. Gender: ……………………………………….....
4. Class: ……………………………………………
5. Ethnic group:
6. Brahmin 1
7. Chhetri 2
8. Newar 3
9. Tamang 4
10. Gurung 5
11. Others (specify)……….... 6
12. Religion:
13. Hindu 1
14. Buddhist 2
15. Christian 3
16. Muslim 4
17. Others (specify)…………… 5

**GROUP B: Household characteristics**

1. How many members do you have in your family? ………………………………...
2. What is the education level of your mother?

a) Literate 1

b) Illiterate 2

c) Primary level 3

d) Secondary level 4

e) College/ University 5

1. What is the education level of your father?

a) Literate 1

b) Illiterate 2

c) Primary level 3

d) Secondary level 4

e) College/ University 5

1. What is the education level of your mother?

a) Literate 1

b) Illiterate 2

c) Primary level 3

d) Secondary level 4

e) College/ University 5

1. What is your father’s occupation?

a) Agriculture 1

b) Service 2

c) Business 3

d) Wage/labour 4

e) Housework 5

f) Others (specify)…………………………. 6

1. What is your mother’s occupation?

a) Agriculture 1

b) Service 2

c) Business 3

d) Wage/labour 4

e) Housework 5

f) Others (specify)…………………………. 6

**GROUP C: Knowledge and practice on LF and MDA**

1. Did you share about LF and MDA that you learn with anyone?
2. Yes 1
3. No 2
4. If yes, with whom did you share?
5. Family member 1
6. Friends 2
7. Neighbour 3
8. Others (specify)………………………………………………………………. 4
9. What kind of microorganism causes Lymphatic Filariasis?
10. Parasite 1
11. Viruses 2
12. Bacteria 3
13. Fungi 4
14. Don’t Know 5
15. How is Lymphatic Filariasis spread? (Multiple choice)
16. Airborne (sneezing, coughing, etc.) 1
17. Food 2
18. Genetically inherited 3
19. Mosquitoes 4
20. Witch 5
21. Don’t know 6
22. Others (specify)…………………. 7
23. Lymphatic Filariasis is mostly seen in countries that are in the tropical or sub-tropical locations.
24. Yes 1
25. No 2
26. Don’t know 3
27. An individual can contract Lymphatic Filariasis after just one bite
28. Yes 1
29. No 2
30. What are the preventive measures of Lymphatic Filariasis? (Multiple choice)
31. Mosquito net 1
32. Fumigation 2
33. Isolation from patient of LF 3
34. Sanitation 4
35. Don’t know 5
36. Others(specify)………………………………………………………………….6
37. There is no treatment for Lymphatic Filariasis
38. Yes 1
39. No 2
40. Don’t know 3
41. The signs and symptoms of Lymphatic Filariasis include: (Multiple Choice)
42. Body aches and pain 1
43. Fever 2
44. Lymphatic edema 3
45. Hydrocele 4
46. All of the above 5
47. Don’t Know 6
48. Once a person has the swelling of limbs, he/she cannot control the progression of the condition
49. Yes 1
50. No 2
51. The medicines used in MDA prevent LF
52. Yes 1
53. No 2
54. Did you take MDA drugs in last round of MDA campaign in the month of Feb-March? **(If yes, skip to Ques. 14)**
55. Yes 1
56. No 2
57. Don’t know 3
58. If no, why didn’t you take MDA drugs? (Multiple Choice)
59. Not at home 1
60. Fear of adverse events 2
61. Health worker did not deliver drugs 3
62. Parent’s didn’t allow 4
63. Dislike medicine 5
64. Don’t have any reason 6
65. If yes, did health worker distributing MDA drugs adequately provided the information about drugs and disease??
66. Yes 1
67. No 2
68. I don’t know 3
69. Have you had any side effects after you took the drugs?
70. Yes 1
71. No 2
72. If yes, what were the side effects? ( Multiple choice)
73. Dizziness 1
74. Headache 2
75. Stomach ache
76. Nausea and vomiting 3
77. Fever 4
78. Others (specify)……………………………. 5
79. Did you have any treatment of side effect after?
80. Yes 1
81. No 2
82. No response 3

**Thank You!**
